# Thermal and Mechanical Interfacial Behaviors of Graphene Oxide-Reinforced Epoxy Composites Cured by Thermal Latent Catalyst

**DOI:** 10.3390/ma12081354

**Published:** 2019-04-25

**Authors:** Shahina Riaz, Soo-Jin Park

**Affiliations:** Department of Chemistry, Inha University, Incheon 402-751, Korea; shahinaawan519@gmail.com

**Keywords:** epoxy, composites, graphene, thermal stability, latent catalyst, cationic curing

## Abstract

A series of composites was prepared from a diglycidyl ether of bisphenol A (DGEBA) with different graphene filler contents to improve their mechanical performance and thermal stability. Graphene oxide (GO) and GO modified with hexamethylene tetraamine (HMTA) were selected as reinforcing agents. As a latent cationic initiator and curing agent, N-benzylepyrizinium hexafluoroantimonate (N-BPH) was used. The effect of fillers and their contents on the mechanical properties and thermal stability of the composites were studied. Fracture toughness improved by 23% and 40%, and fracture energy was enhanced by 1.94- and 2.27-fold, for the composites containing 0.04 wt.% GO and HMTA-GO, respectively. The gradual increase in fracture toughness at higher filler contents was attributed to both crack deflection and pinning mechanisms. Maximum thermal stability in the composites was achieved by using up to 0.1 wt.% graphene fillers.

## 1. Introduction

Epoxides containing one or more epoxy groups at the end, middle, or center of a molecular chain are considered reactive species which can readily react with curing agents to produce a three-dimensional polymer structure. These thermosetting polymer structures are widely used in various applications such as paints, coatings, and adhesives due to their high modulus strength, excellent chemical and corrosion resistivities, excellent dimensional stability, good adhesive properties, and low degree of shrinking during the curing process [1,2,3]. However, epoxy resins exhibit some undesirable properties such as brittleness and poor crack resistance which limit their applicability [4]. A commonly used method to overcome these shortcomings of epoxy resins is the introduction of organic or inorganic nano- or micro-sized fillers to the matrix [5,6]. Among these commonly used fillers, carbon nanomaterials (CNMs) such as carbon nanotubes (CNTs), carbon nanofibers, and graphene are materials of great interest for decreasing the brittleness of epoxy resins as well as slightly increasing their thermal stability [7,8,9,10,11,12,13,14]. Graphene with sp^2^-bonded carbon atoms is now the most attractive nanofiller in materials science due to its unusual thermal, electrical, and mechanical properties [14,15,16,17]. Due to these fundamental properties, many applications have been proposed for graphene and its various chemically modified forms. Among these, chemically modified graphene obtained from graphite oxide (GO) has attracted much attention as a filler for polymer nanocomposites such as polyethylene glycol, polyethylene vinyl acetate, and epoxy, due to its convenient processing in liquids. In previous studies it was proven that the addition of GO to polymer matrix can significantly improve the mechanical properties compared to graphene [18,19,20,21,22,23,24]. 

The agglomeration of graphene within polymer matrices, due to its low compatibility with polymers, limits its application as a mechanical reinforcing material. To overcome these problems many strategies had been developed to chemically or physically modify graphene [25,26,27,28,29]. Previous studies have shown that the presence of amino groups on the surface of graphene increased its adhesion with epoxy resins and also improved its thermal and mechanical characteristics [30] Ribeiro et al. [31] prepared a tetraethylenepentamine (TEPA)-functionalized GO epoxy nanocomposite which resulted in homogeneous dispersion. Measurements showed an increase of 72% and 143% in elastic modulus and hardness, respectively, for the composite containing 0.5 wt.% GO-TEPA in the epoxy matrix. For the same composite, improved thermal properties and a 103% increase in thermal conductivity were also reported. Since many industrial applications of graphene are still limited by its relatively high cost, the improvements at low loadings of graphene oxide provide opportunities to produce cost effective epoxy/graphene oxide nanocomposites.

Curing agents (CA) are necessary for the initiation of ring-opening polymerization of epoxies to convert them to a three-dimensional network. The properties of epoxies largely depend on the chemical structure of the CA and curing of epoxies via homopolymerization has certain advantages and broadens the possible applications of such polymeric materials [32,33]. Cationic initiators, which remain inactive under normal environmental conditions and become active by external stimulation by heat or photoirradiation, have been synthesized and their importance has been recognized in the fields of organic and polymer synthesis [34,35,36]. Hence, the effective control of curing and initiation by heating provides desirable advantages in terms of stability during handling and storage, which leads towards energy-saving and environmentally friendly processing in comparison to materials incorporating conventional CAs like anhydrides and amines. In practical applications, heating is much more convenient than photoirradiation. The irradiation area depends upon the source of irradiation, so homogeneous irradiation is often difficult to attain. Thick samples and shadowed areas in particular are difficult to completely cure by photoirradiation [37].

Hexamethylenetetramine (HMTA) is a heterocyclic polar organic compound with a cage-like structure bearing four tertiary amine groups. It is considered to be a green and versatile organic compound for use in organic synthesis. If this tertiary amine group is introduced into carbon fillers, the wettability and interfacial interaction of the filler material with the epoxy matrix could be increased significantly. HMTA can also be used as a curing and toughening agent in epoxy matrices [38]. 

Herein we report the surface modification of GO sheets with HMTA. The modified GO sheets (HMTA-GO) were then used as reinforcing agents in epoxy composites. The nanocomposites were prepared using a solvent-free method in which the nanofillers were introduced to the epoxy resin through ball milling, yielding a homogeneous dispersion. N-benzylepyrizinium hexafluoroantimonate (N-BPH) was used as a thermally latent catalyst. The filler contents were kept below 0.2 wt.%. The influences of GO and HMTA-GO on the thermal and mechanical performance of the epoxy composites were studied. A commercially available diglycidyl ether of bisphenol A (DGEBA) was used as a base resin, and the thermally latent catalyst N-BPH was synthesized following a previously reported procedure [39].

## 2. Materials and Methods

### 2.1. Materials

DGEBA with an epoxide equivalent weight of 185–190 g eq^−1^ and density of ~1.16 g/cm^3^ at 25 °C was purchased from Kukdo Chemical Co., Seoul, Korea. Benzyl bromide, pyrazine, hexamethylene tetraamine (HMTA), and sodium hexafluoroantimonate were supplied by Aldrich Chemical Co., Seoul, Korea, Acetonitrile, methanol, and diethyl ether were purchased from Daejung Chemical and Metals Co., Ansan, Korea, Phosphoric acid (H_3_PO_4_), hydrogen peroxide (as 30% H_2_O_2_ solution in water), and sulfuric acid (H_2_SO_4_) were supplied by Duksan Pure Chemicals, Ansan, Korea.

### 2.2. Synthesis of GO and HMTA-GO

Graphene oxide was synthesized by a modified Hummer’s method [4,14]. For the preparation of HMTA-GO samples, 1 g of GO was added to deionized water and the dispersion was sonicated for 30 min. Then, 50 mL aqueous of a solution of HMTA (5 g) was added to the GO dispersion with strong stirring, and the mixture was left to react at 80 °C for 72 h. The resultant mixture was filtered, and the black solid obtained was washed with deionized water and ethanol to remove unreacted HMTA and then freeze dried at 70 °C and 0.045 mbr for 70 h.

### 2.3. Synthesis of Epoxy Composites

For the preparation of composite samples, the required amounts of GO and HMTA-GO were first dispersed in acetone and sonicated for 20 min. After sonication, the GO and HMTA-GO dispersions were added to the epoxy resin and stirred at 60 °C until a homogenous mixture was obtained. To further enhance the dispersion of the fillers in the epoxy, the mixture was ball milled for 4 h at 200 rpm to exfoliate agglomerates of the graphene fillers. The resulting mixture was placed on a hot plate at 80 °C for 24 h to evaporate the solvent. N-BPH was then added, and the formulation was stirred at 70 °C for 1 h. All samples were prepared on a mass basis by adding 3 parts per hundred of resin (phr) of N-BPH to DGEBA, and the filler in 0.04, 0.06, 0.08, 0.1, and 0.2 wt.%. The sample mixtures were then degassed in a vacuum oven at 60 ℃ to remove bubbles. Finally, the mixtures were poured into aluminum molds and cured at 80, 120, 170, 200 and 230 °C for 2 h at each temperature. The chemical structures of HMTA, N-BPH, and DGEBA are shown in Figure S1.

### 2.4. Characterization

The structures and morphologies of GO and HMTA-GO were determined by field emission transmission electron microscopy (FE-TEM; JEM-2100F, JEOL, Tokyo, Japan). Thermogravimetric analysis (TGA; TG-209 F3 NETZSCH, Bavarian, Germany) was used to investigate the thermal stabilities of GO, HMTA-GO, and the composites. Typical conditions involved a temperature range of 30–600 °C and a heating rate of 10 °C /min under N_2_ atmosphere. Fourier-transform infrared spectroscopy (FTIR; Jesco PS-4000, JESCO, Easton, USA) was performed at room temperature over the wavelength range of 4000–500 cm^−1^. Raman analysis was carried out at room temperature using a Raman spectroscope (HORIBA) with a 512 nm laser light. Cross-sectional images of the synthesized samples were obtained by high-resolution scanning electron microscopy (HR-SEM; SU-8010, Hitachi, Ltd., Tokyo, Japan). X-ray photoelectron spectroscopy (XPS; ESCA LAB MK-II, Scientific Co., Netherlands) was carried out for surface chemistry analysis of the graphene fillers. Monochromatic Mg Kα X-ray radiation was used as the excitation source. X-ray diffraction (XRD, D2 Phaser, Bruker, Panalytical Incorporated, Netherlands) was performed at room temperature with a Cu target, operated at 40 kV and 30 mA. The XRD patterns were obtained from 10 to 60° (2θ) at a rate of 5° min^−1^. Elemental analysis of N-BPH was performed using an EA1112 elemental analyzer (ThermoFisher scientific, Seoul, Korea). Cure behavior of composite samples were investigated by differential scanning spectroscopy (DSC-404 F1, NETZSCH, Bavarian, Germany).

Fracture toughness (*K_IC_*, critical stress intensity factor; and *G_IC_*, critical strain energy release) and flexural properties of the composites were measured according to ASTM-D5045 using single-edge notch specimens in a three-point bending flexural test with a universal testing machine (Instron Model 1125 mechanical tester, Instron, Norwood, MA, USA).

The *K_IC_* of the cured specimen was calculated as follows:

(1) $$K_{IC}=\frac{P_{\max}}{bd^{{}^{1/2}}}f(x)$$

*x* = *a*/*d*

where *P_max_, d, b, f(x),* and *a* are the critical load for crack propagation, width of the specimen, thickness of the specimen, geometrical factor given by Equation (2), and pre-crack length, respectively [40].

(2) $$f(x)=6x^{1/2}\frac{\left[1.99-x(1-x)(2.15-3.93x+2.7x^{2})\right]}{2(1+2x){\left(1-x\right)}^{3/2}}$$

The *G_1C_* of the composite sample was calculated using the following equation:

(3) $$G_{1C}=\frac{\left(1-\nu^{2}\right)}{E}K_{IC}^{2}$$

where *ν* is the Poisson’s ratio for epoxy (0.348) and *E* is young’s modulus as determined from the fracture toughness test. The flexural properties of composite samples were measured according to ASTM-D790 using an Instron Model 1125 mechanical tester (Instron, Norwood, MA, USA) [41]. Five experimental measurements were recorded and averaged for each of the mechanical properties.

## 3. Results

### 3.1. Characterization of Filler

**Figure 1:**
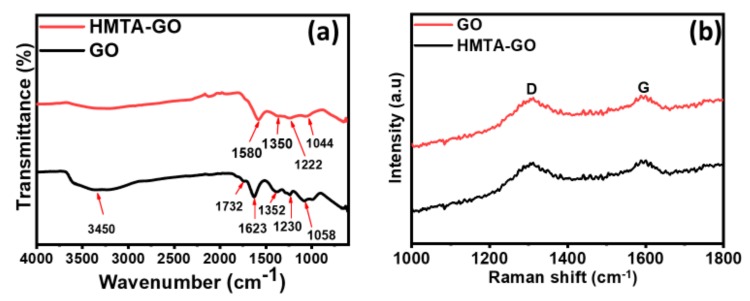
(**a**) FTIR (**b**) Raman spectra of GO and HMTA-GO.

FTIR studies were carried out to investigate the functionalization of both GO and HMTA-GO. As shown in Figure 1a, many oxygen-containing functional groups were observed in the structure of GO. The characteristic band in the range of 3000–3500 cm^−1^ corresponds to OH stretching [42], the band at 1732 cm^−1^ is related to carbonyl stretching, the band at 1230 cm^−1^ corresponds to the epoxide functional group [43], and COH/COC stretching vibrations were observed at 1352 cm^−1^ and 1058 cm^−1^ [44]. The C=C stretching of the unoxidized graphitic domain due to skeletal vibrations was observed at 1623 cm^−1^. In the FTIR spectrum of HMTA-GO, significant shifts of the OH and COH/COC stretching bands to lower wavenumbers were observed relative to their positions in GO, which indicates strong interactions between these groups and HMTA. HMTA has a symmetric nature, so it can react simultaneously with many sites of the GO structure. The principal interaction between HMTA and GO is likely via hydrogen bonding [42,45]. 

Raman spectroscopy is another important technique to evaluate the structural changes in the prepared samples. Figure 1b shows the Raman spectra of GO and HMTA-GO. As expected, the spectrum of GO exhibits two characteristics peaks for G band at 1329 cm^−1^ and for D band at 1587 cm^−1^. The G band could be ascribed to all the sp^2^ carbon atoms describing the in-plane vibration of sp^2^ bonded carbon atoms while, the D band suggests presence of sp^3^ defects [46]. It could be observed that the G peak of HMTA-GO is moved to1584 cm^−1^ which is an indicative of the successful functionalization of GO.

**Figure 2:**
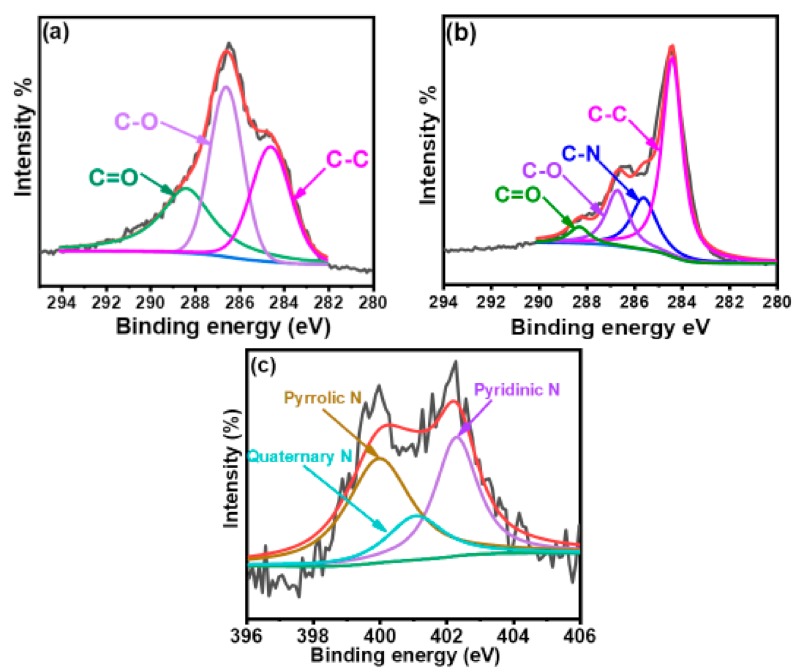
High resolution XPS spectra of C1s for; (**a**)GO (**b**) HMTA-GO (**c**) N1s for HMTA-GO.

Figure XPS survey spectra were measured to further investigate the chemical composition of GO before and after its modification with HMTA. Figure 2a,b shows high-resolution C1s XPS spectrum for the graphene fillers; the relative peak areas (in %), full widths at half maximum (FWHM) of different C1s peaks, and atomic percentage of C, O, and N in GO and HMTA-GO are summarized in Tables S1 and S2. From the survey scans of GO and HMTA-GO (Figure S2), a new peak at 401 eV (N1s) was observed for HMTA-GO which was absent in pure GO, which confirms the modification of GO with HMTA. In Figure 2a, it can be seen that GO exhibited a considerable degree of oxidation, as indicated by the C:O atomic ratio of 59.75%:40.25%. The C1s spectrum of GO was deconvoluted into three different peaks corresponding to the carbon atoms in different functional groups. The characteristic peaks at 284.6 eV (C–C), 286.6 eV (C–O), and 288.6 eV (O–C=O) were attributed to unoxidized graphitic carbon, carbon atoms in hydroxyl and epoxy/ether groups, and carboxyl groups, respectively [47]. In the C1s spectra of HMTA-GO (Figure 2b), a new peak was observed at 285.5 eV, which confirms the successful attachment of HMTA molecules to the GO sheets. Figure 2c shows the N1s spectra for HMTA-GO. N1s spectra of HMTA-GO can be deconvoluted into three peaks which corresponds to pyrrolic N (400 eV), quaternary N (401 eV) and N oxides of pyridinic N (402.3 eV) [48]. 

**Figure 3:**
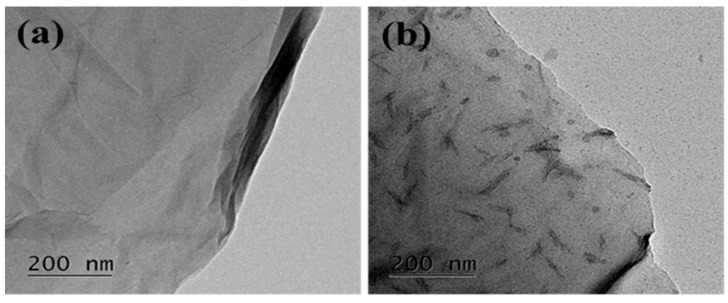
FE-TEM images of (**a**) GO and (**b**) HMTA-GO.

The morphologies of GO and HMTA-GO were observed using HR-SEM. Figure S3a,b shows HR-SEM images of GO and HMTA-GO, respectively. Figure S3 shows that GO exhibit thin morphology, after functionalization more irregular and rough texture was observed. The FE-TEM images of GO and HMTA-GO are shown in Figure 3a,b, respectively. In this Figure, GO exhibited a wrinkled and transparent morphology; the surfaces of the GO sheets were smooth, so the wrinkled morphology was due to the flexibility of the 2D sheets. The ultra-thin nature of GO sheets make them almost invisible. GO exhibited a thin, sheet-like morphology, while HMTA-GO exhibited an irregular and rough surface which can be attributed to structural defects. The rough and slightly fuzzy surface of HMTA-GO in the FE-TEM images, could be attributed to the attachment of HMTA to GO sheets. 

**Figure 4:**
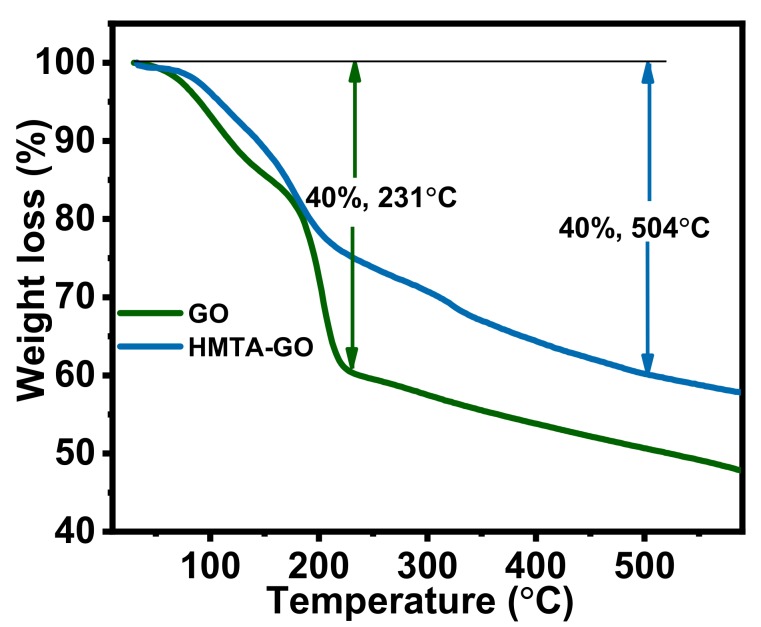
TGA curves for GO and HMTA-GO.

TGA is among the most commonly used techniques to investigate the decomposition of polymers at various temperatures, and the thermal stability of materials in general. The thermal stabilities of the graphene fillers were assessed under nitrogen atmosphere at a heating rate of 10 °C/min. Figure 4 shows TGA curves for GO and HMTA-GO. The curves reveal that GO experienced a mass loss of 40% at 231 °C, while the 40% mass loss in HMTA-GO was at 504 °C. In the TGA profile, between 28 °C and 150 °C, GO experienced a loss in mass of 14.5%, which was possibly due to the desorption of physically absorbed water molecules. The thermal degradation profile of HMTA-GO exhibited a three-step degradation behavior: the weight loss between 28 °C and 119 °C was attributed to desorption of physically absorbed water molecules, and the weight loss at higher temperatures was attributed to loss of the HMTA molecules. HMTA sublimates between 285 and 295 °C [49], and it can therefore be assumed that all HMTA molecules left the interlayer spaces within the temperature range shown in the TGA curves. Hence, the presence of HMTA molecules in the GO matrix, which interact strongly with GO, remarkably increased the thermal stability of GO. 

### 3.2. XRD of Epoxy Composites

XRD is an important technique to investigate the degree of exfoliation of GO in the composite materials. Figure S4 shows the XRD spectrum of neat epoxy and its composites. Neat epoxy exhibits a wide diffraction from 12–28° which is associated with the scattering of cured epoxy molecules indicating the amorphous nature of epoxy. All the epoxy composites exhibit similar diffraction patterns as the neat epoxy demonstrating that the GO nanofillers are highly exfoliated in epoxy matrix [50].

### 3.3. Thermal Stability and Degradation Kinetics of Epoxy Composites

**Figure 5:**
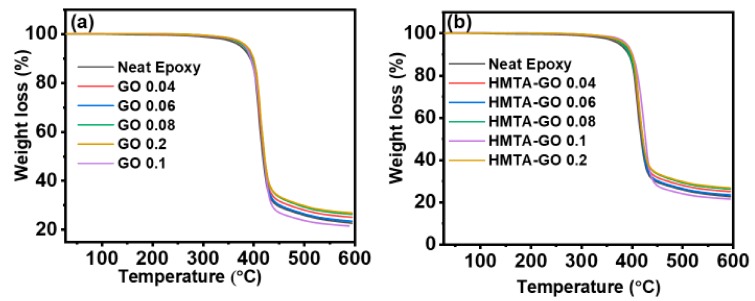
TGA curves for epoxy composites (**a**) GO (**b**) HMTA-GO.

Figure 5a,b shows the TGA curves for the GO/epoxy and HMTA-GO/epoxy nanocomposites; all TGA curves appeared to be similar regardless to the filler contents. From the TGA curves, it was deduced that insertion of fillers into the epoxy did not change the decomposition mechanism of the epoxy matrix; single-step decompositions corresponding to the decomposition of macromolecular chains were observed. All the specimens exhibited decomposition behaviors like that of the epoxy matrix. However, a very careful observation led us to suggest that HMTA-GO epoxy composites exhibit higher thermal stability than GO/epoxy composites, and that the thermal stability of the composites exhibits a positive relationship with increasing filler content. GO showed a less-significant effect on the thermal stability of the epoxy composites than HMTA-GO which could be attributed to the weaker interaction between GO and epoxy than that of HMTA functional groups attached to GO [51,52]. To investigate the inherent thermal stability of the composites, the integral procedure decomposition temperature (IPDT) proposed by Doyle was calculated using the following Equations [53]:

(4) $$IPDT\left({}^{\circ }C\right)=A^{*}.K^{*}\left(T_{f}-T_{i}\right)+T_{i}$$

where

(5) $$A^{*}=\frac{X_{1}+X_{2}}{X_{1}+X_{2}+X_{3}}$$

and

(6) $$K^{*}=\frac{X_{1}+X_{2}}{X_{1}}$$

In these equations, A* is the area ratio of the total experimental curve and total TGA thermogram, and *T_i_* and *T_f_* are the initial and final experimental temperatures, respectively. Figure S4 shows a schematic illustration of *X_1_, X_2_*, and *X_3_* for calculations of A* and K*. Table 1 shows the IPDT and initial decomposition temperatures (IDT) at 5% weight loss for the epoxy composites; the IPDT values varied with filler content. The IPDT was found to increase linearly with increasing GO and HMTA-GO contents, which confirms the improved thermal stability of the composites.

The degradation kinetics and other kinetic parameters of the epoxy composites were calculated using the Horowitz-Metzger integral kinetics method (HMIK) [54]. The HMIK method determines the activation energy (*E_a_*) of decomposition at a single heating rate [55]. In this study, the TGA thermograms obtained at the heating rate of 10 °C/min were used to calculate the *E_a_*_._ The HM equation is shown as follows:

(7) $$\ln\left\{\ln\left(\frac{1}{1-\alpha }\right)\right\}=\frac{E_{a}\theta }{RT_{e}^{2}}$$

where *α, R,* and *E_a_* are heating rate, universal gas constant, and pyrolysis activation energy, respectively, *θ* = *T* − *T_e_*, *T* is the temperature at time *t*, *T_e_* is the temperature at which *w_o_*/*w_t_* = 1/*e* (1/*e* = 0.368), *w_o_* is the initial weight, and *w_t_* is the weight of the sample at time *t.* The plot of ln(ln(1/1 − *α*)) against *θ* should give a straight line. The regression analysis provided the slope, constants, and *R^2^* values, and the slope equation was then used to calculate the *E_a_* of decomposition for the composite samples; Figure S5 shows the plot of ln(ln(1/1 − *α*)) against *θ*. Considering the results tabulated in Table 2, it was evident that the kinetic parameters are affected upon increasing filler content. The pyrolysis *E_a_* of the samples increased upon increasing the filler content from 0.04 wt.% to 0.1 wt.%. This increase in *E_a_* was more pronounced in the HMTA-GO composites samples due to strong interactions between the modified GO sheets and the matrix.

### 3.4. Mechanical Properties of Epoxy Composites

**Figure 6:**
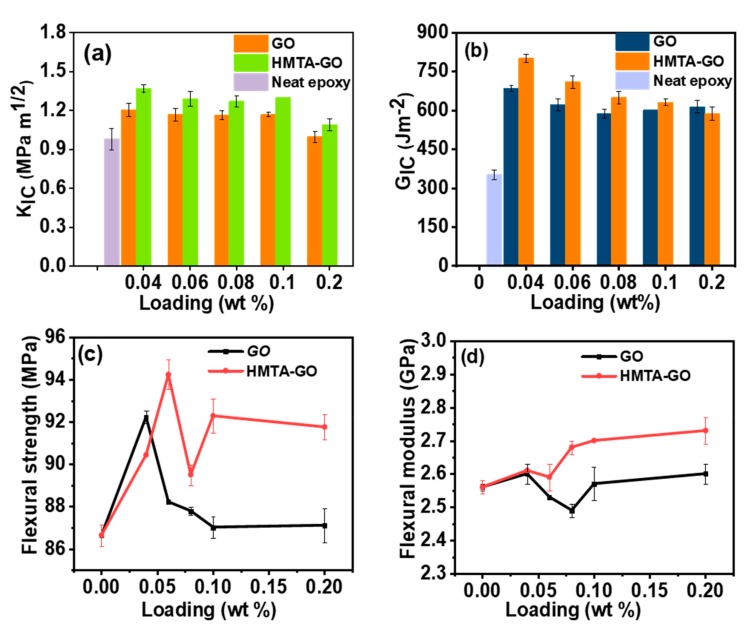
(**a**) Fracture toughness, (**b**) Fracture energy, (**c**) Flexural strength, and (**d**) Flexural modulus plots of epoxy composites.

The model-I *K_IC_* and *G_IC_* of the epoxy composites, as measured by three-point bending tests, were plotted as functions of the filler contents; the results are shown in Figure 6a,b, respectively. The neat epoxy exhibited a *K_IC_* value of ≈ 0.98 MPa m^1/2^, which correlates well with values published in the literature [56,57]. Addition of both GO and HMTA-GO at 0.04 wt.% loadings caused sharp increases in the *K_IC_* of the epoxy composites, to 1.40 MPa m^1/2^ and 1.54 MPa m^1/2^, respectively. The *K_IC_* of both GO and HMTA-GO epoxy composites shows sharp decrease upon increasing the loadings of GO and HMTA-GO at 0.06 wt.% and 0.08 wt.%, respectively, and then increased gradually higher loadings. 

The maximum enhancements to *K_IC_* in the GO and HMTA-GO epoxy composites were 23% and 40% increases, respectively. During the curing of the composites, the functional groups of GO and HMTA-GO facilitate interfacial interactions between the fillers and the epoxy, resulting in increased energy dissipation for microcrack formation. Due to this phenomenon, the epoxy composites with graphene fillers exhibited greater fracture toughness than that of the neat epoxy. Polymer failure is a complex process which involves the loss of structural integrity at both micro- and macroscopic levels under deforming conditions [58]. The GO and HMTA-GO epoxy composites showed maximum improvements to *G_IC_* of 1.94- and 2.27-fold at 0.04 wt.% loadings of the filler. Here, the better result obtained with HMTA-GO could be attributed to its better dispersion than that of GO, resulting in enhanced interfacial interactions between the filler and the matrix. In previous a study, it was assumed that a crack deflection mechanism is responsible for the higher fracture toughness of epoxy composites at lower graphene loadings, and a decrease in *K_IC_* at higher graphene loadings was attributed to graphene aggregation [59].

In present study, the *K_IC_* first increased with small graphene loadings, which could be attributed to the microcrack formation, and then decreased before gradually increasing at higher graphene loadings, as crack pinning and crack deflection were more influential in the toughening of the composites at smaller graphene loadings. If the microcrack zones are in close proximity to each other, then the coalescence of the microcracks facilitates propagation of major cracks [60]. The increases in *K_IC_* at higher graphene contents were less drastic due to coalescence of microcracks, which causes the formation of primary cracks. Hence, as shown in this study, with an optimized surface modification, the toughening effect of graphene-based materials in epoxy resins can be further improved. The flexural strength and flexural modulus plots of the epoxy composites are shown in Figure 6c,d, respectively. In this figure, it can be observed that the flexural moduli and strengths of the composites containing graphene fillers varied as filler content was varied between 0.04 wt.% and 0.2 wt.% The less-significant effect of GO on the flexural modulus of the composites (in comparison to that of HMTA-GO) is due to the weak interfacial interactions between GO and the epoxy, and the aggregation of GO sheets within the epoxy matrix. HMTA-GO induced a slight increase in the moduli of the composites, which was attributed to the enhanced interfacial interaction between the epoxy and HMTA-GO as the amine-rich environment of HMTA, can form covalent bonding between the GO sheets and the matrix during the curing procedure [31]. Consequently, the interfacial interaction between GO and matrix becomes stronger.

**Figure 7:**
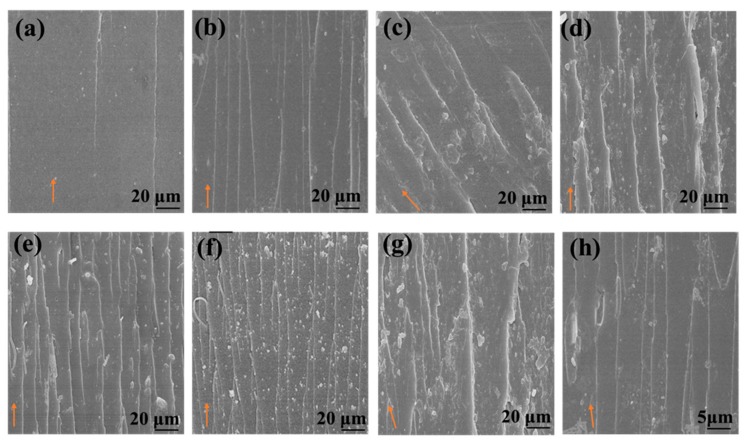
SEM images of epoxy composites with graphene fillers in wt. % (**a**) Neat epoxy (**b**) GO 0.04 (**c**) GO0.08 (**d**) GO 0.2 (**e**) HMTA-GO 0.04 (**f**) HMTA-GO 0.08 (**g**) HMTA-GO 0.2. h is the higher magnification image of HMTA-GO 0.2 (arrows indicate direction of crack propagation).

The fracture mechanism in the epoxy composites was further investigated by SEM. As shown in Figure 7a, due to the intrinsically brittle nature of the epoxy matrix, it emerged with a very smooth fractured surface, and almost no remarkable events were observed during the crack propagation process because pristine epoxy shows catastrophic fracture behavior due to the absence of energy absorbing events. On the other hand, in a crosslinked structure, the release of a rubbery phase during debonding from the epoxy matrix can dissipate a significant amount of energy, resulting in increased fracture toughness of the epoxy. However, in epoxies filled with rigid fillers, the toughening mechanism involves plastic yielding of the epoxy matrix around the rigid fillers, followed by particle interference and formation of voids, during a crack propagation event [61]. The surface roughness increased with increasing filler content due to increased crack deflection as well as pinning and coalescence of microcracks. In the case of the HMTA-GO epoxy composites, the fracture surface exhibited deeper voids and rough surface, indicating that modified GO interacts strongly with the epoxy matrix. The presence of strong stretched lines in the epoxy composites with higher filler contents (Figure 7g) indicates that the epoxy matrix was pulled out from the graphene fillers during crack propagation, generating stretched lines in the direction of propagation [62].

## 4. Conclusions

In this study, graphene oxide (GO) sheets and GO modified with hexamethylene tetraamine (HMTA) were used as fillers to obtain a series of epoxy composites. TEM images confirmed the attachment of HMTA to the GO surface. Filler contents ranging from 0.04 to 0.2 wt.% were incorporated into the epoxy resin through ball milling to obtain homogeneous dispersions. A maximum toughening effect was observed at lower filler loadings, for both type of graphene composites; a 23% improvement in *K_IC_* and 1.94-fold enhancement in *G_IC_* were achieved at 0.04 wt.% GO loading in the GO epoxy composite, while a 40% enhancement in *K_IC_* and 2.27-fold increase in *G_IC_* were achieved with the same filler loading in the HMTA-GO epoxy composite. At higher filler loadings, microcrack coalescence facilitated crack propagation, resulting in decreased fracture toughness. Gradual increase in fracture toughness at higher filler contents were attributed to both crack deflection and pinning mechanisms. The thermal stability of the composites also varied with filler content, with maximum thermal stability achieved at 0.1 wt.% loading for both graphene fillers; this was because GO and modified GO sheets insulate the underlying material and decrease its rate of decomposition.

## Figures and Tables

**Table 1:** Kinetic thermodynamic parameters of epoxy composites obtained by TGA thermograms.

Samples	A*	K*	A*. K*	IPDT (°C)	IDT (°C) (5% Weight Loss)	T_e_ (°C)
Neat Epoxy	0.703	1.597	1.123	664	381	428
GO 0.04 HMTA-GO 0.04	0.705	1.601	1.129	668	382	428
	0.708	1.605	1.136	672	383	440
GO 0.06 HMTA-GO 0.06	0.706	1.603	1.132	669	382	431
	0.709	1.609	1.141	675	384	442
GO 0.08 HMTA-GO 0.08	0.709	1.613	1.144	676	383	431
	0.711	1.614	1.148	678	385	446
GO 0.1 HMTA-GO 0.1	0.711	1.615	1.148	679	387	439
	0.715	1.618	1.157	684	390	450
GO 0.2 HMTA-GO 0.2	0.714	1.615	1.153	682	387	431
	0.719	1.619	1.164	688	391	448

T_e_ = decomposition temperature.

**Table 2:** Kinetic thermodynamic parameters of epoxy composites calculated by using Horowitz-Metzger method.

Samples	E (J/mol)	R^2^
Neat epoxy	127	0.998
GO 0.04 HMTA-GO 0.04	132	0.998
	136	0.997
GO 0.06 HMTA-GO 0.06	135	0.994
	141	0.996
GO 0.08 HMTA-GO 0.08	143	0.993
	161	0.999
GO 0.1 HMTA-GO 0.1	152	0.983
	179	0.996
GO 0.2 HMTA-GO 0.2	147	0.997
	162	0.997

## Supplementary Materials

The following are available online at https://www.mdpi.com/1996-1944/12/8/1354/s1, Figure S1: Chemical structure of HMTA, N-BPH and DGEBA, Figure S2: XPS survey scan of GO and HMTA-GO, Figure S3. SEM images of (a) GO and (b) HMTA-GO, Figure S4. XRD spectrum of (a) Neat epoxy (b) GO 0.04 (c) GO 0.06 (d) GO 0.08 (e) GO 0.1 (f) GO 0.2 (g)HMTA-GO 0.04 (h) HMTA-GO 0.06 (i) HMTA-GO 0.08 (j) HMTA-GO 0.1 (k) HMTA-GO 0.2, Figure S5. Schematic illustration for calculation of A* and K*, Figure S6. Plot of ln(ln(1/1-α)) versus θ, and Figure S7 DSC curves for epoxy composites. Table S1: Analysis of the deconvoluted C1s peaks obtained from XPS and the FWHM of the different C1s peaks as well as the relative area percentages for GO and HMTA-GO, Table S2: Atomic percentage of C, O and N for GO and HMTA-GO, Table S3: Elemental composition of N-PBH, Table S4. Exothermic peak temperatures of epoxy composites obtained by DSC.
