# Supplementary material for: Thermal and Mechanical Interfacial Behaviors of Graphene Oxide-Reinforced Epoxy Composites Cured by Thermal Latent Catalyst
Source: Materials (Basel). 2019 Apr 25;12(8):1354. doi: 10.3390/ma12081354 (PMC6515424; doi:10.3390/ma12081354)
Supplement: Supplementary file 1 [file materials-12-01354-s001.pdf]

materials

Supplementary Materials

Thermal and Mechanical Interfacial Behaviors of Graphene Oxide-Reinforced Epoxy Composites Cured by Thermal Latent Catalyst

Shahina Riaz and Soo-Jin Park *

Department of Chemistry, Inha University, Incheon 402-751, South Korea; shahinaawan519@gmail.com
* Correspondence: sjpark@inha.ac.kr

Received: 1 April 2019; Accepted: 24 April 2019; Published: 25 April 2019

Table S1. Analysis of the deconvoluted C1s peaks obtained from XPS and the FWHM of the different C1s peaks as well as the relative area percentages for GO and HMTA-GO.

	**Cs1 fitting**			
**Samples**	**Binding energy, (eV)**	**Relative area %,**	**FWHM (eV)**	
	C-C	C-O	C=O	C-N
GO	284.6, 43.1, 1.40	286.6, 50.03,1.40	288.6, 20.32, 1.40	-
HMTA-GO	284.4, 60.25, 1.40	286.6, 11.25, 1.40	288.5, 8.23, 1.40	285.4, 7.01,1.40

FWHM= Full width half maximum.

Table S2. Atomic percentage of C, O and N for GO and HMTA-GO.

**samples**	**C1s %**	**O1s %**	**N1s %**
GO	59.75	40.25	-
HMTA-GO	71.61	24.6	3.78

Table S3. Elemental composition of N-PBH.

**Weight %**		
C contents[a]	H contents[b]	N contents[c]
31.99	2.93	6.89

[a]Carbon contents, [b]Hydrogen contents, [c]Nitrogen contents; determined by elemental analysis.

Table S4. Exothermic peak temperatures of epoxy composites obtained by DSC.

Sample	T_i	T_p
Neat Epoxy	159	191
GO 0.04	157	197
GO 0.2	155	202
HMTA-GO 0.04	152	188
HMTA-GO0.2	156	187

T_i= Initial temperature, T_p= Peak temperature.

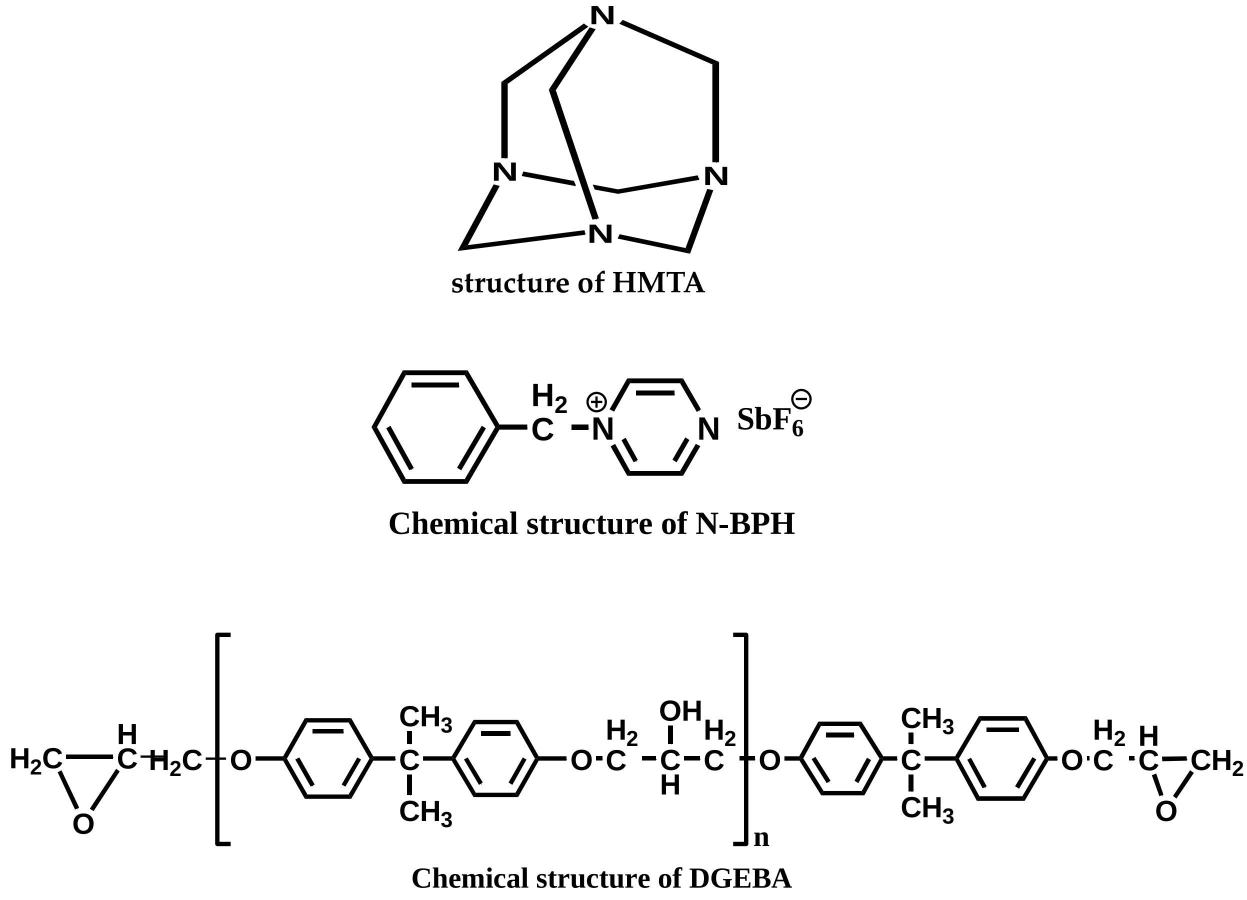

Figure S1. Chemical structure of HMTA, N-BPH and DGEBA.

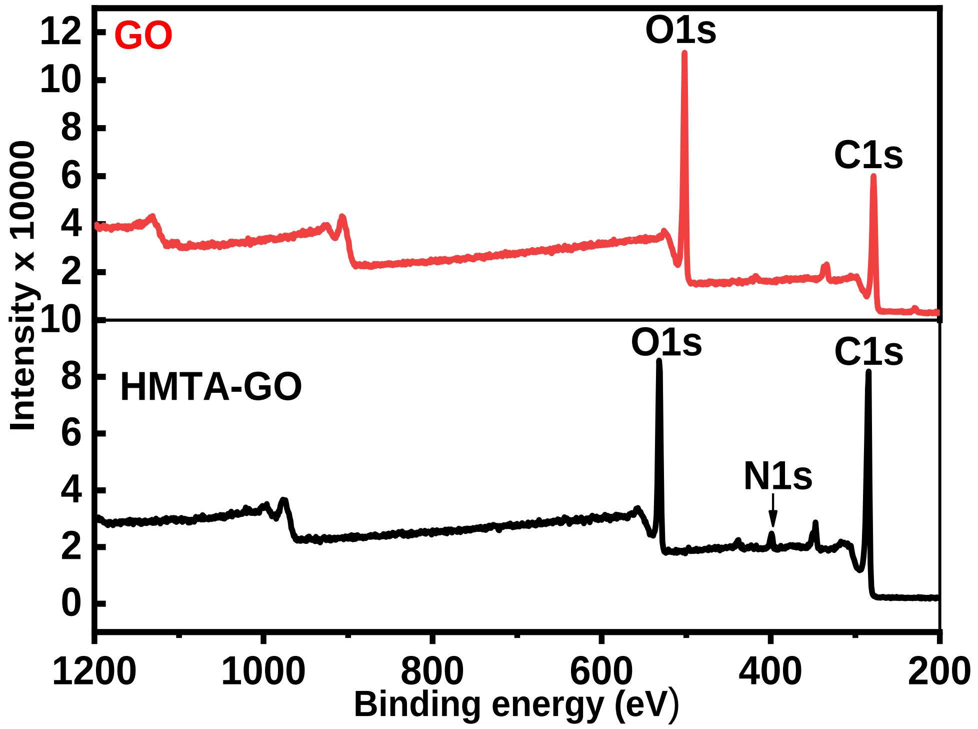

Figure S2. XPS survey scan of GO and HMTA-GO.

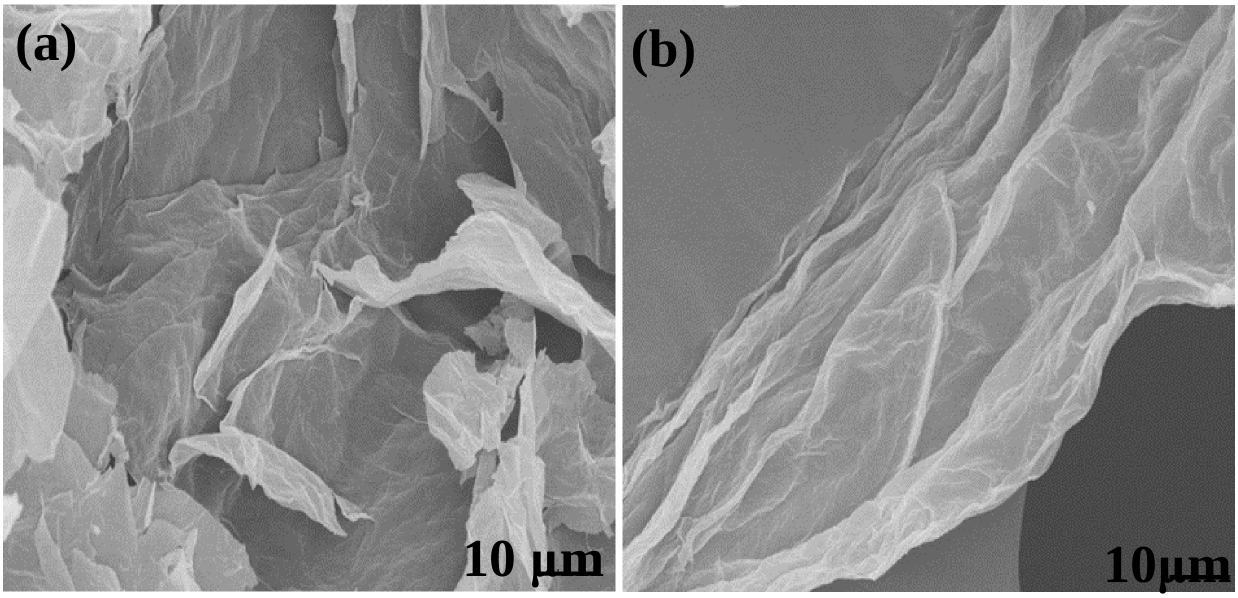

Figure S3. HR-SEM images of (a) GO and (b) HMTA-GO.

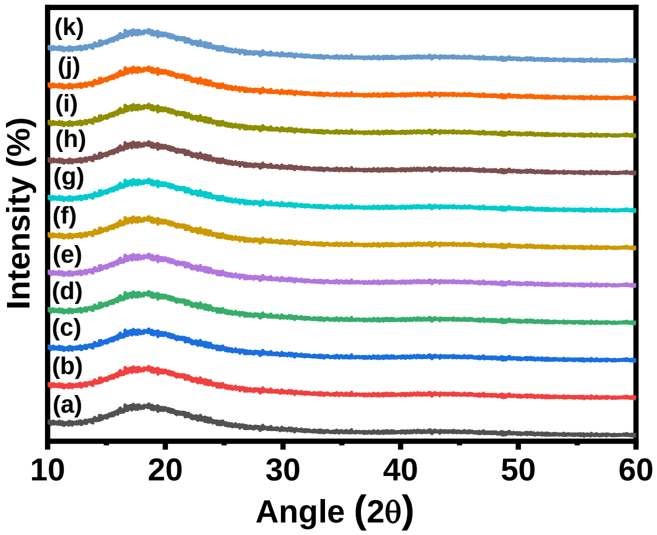

Figure S4. XRD spectrum of (a) Neat epoxy (b) GO 0.04 (c) GO 0.06 (d) GO 0.08 (e) GO 0.1 (f) GO 0.2 (g)HMTA-GO 0.04 (h) HMTA-GO 0.06 (i) HMTA-GO 0.08 (j) HMTA-GO 0.1 (k) HMTA-GO 0.2.

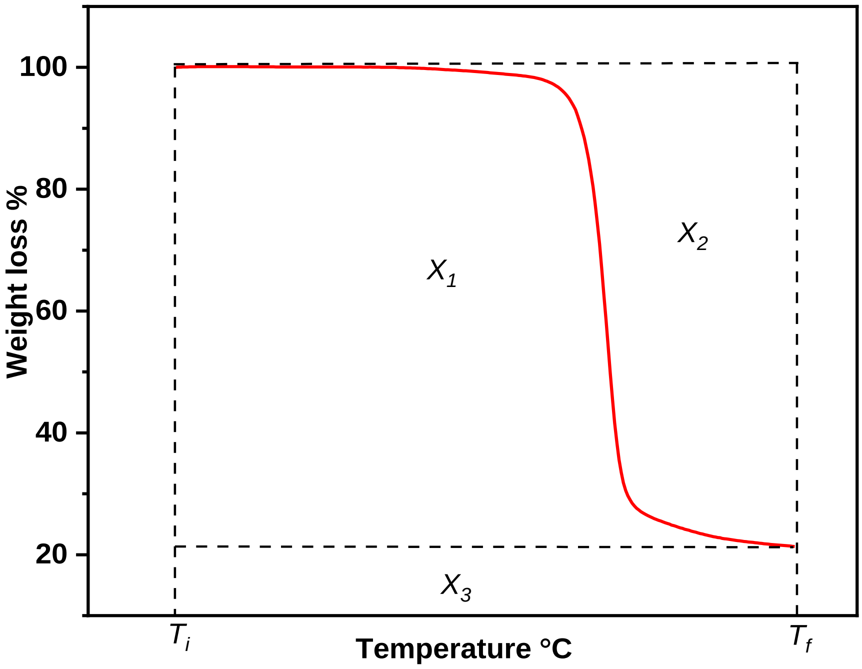

Figure S5. Schematic illustration for calculation of *A** and *K**.

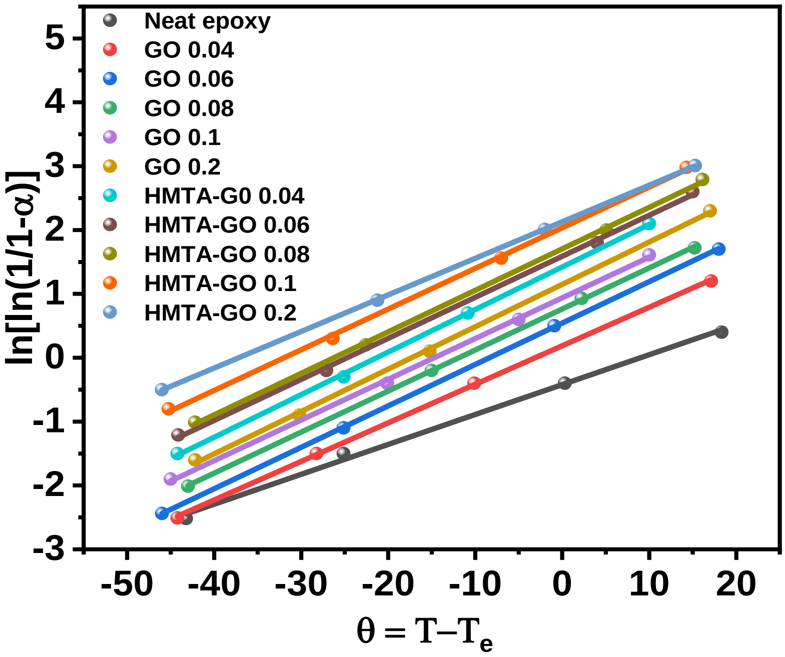

Figure S6. Plot of ln(ln(1/1-α)) versus θ.

Cure behavior of epoxy composites

DSC analysis was carried out to determine the cure behavior of epoxy composites. Figure S6 shows the DSC curves for GO epoxy and HMTA-GO epoxy composites. Table S4 shows the initial curing temperature (T_i) and peak temperature (T_p) for epoxy composites. The exothermic cure reaction occurs between 120 -209 °C These curves exhibit decrease in exothermic peak height of composites containing GO and HMTA-GO compared to neat epoxy indicating enhancement in degree of interaction as well as physical hinderance. The composites containing HMTA-GO exhibit a shift of exothermic peak to lower temperature indicating increase in curing rate for the nanocomposites [1].

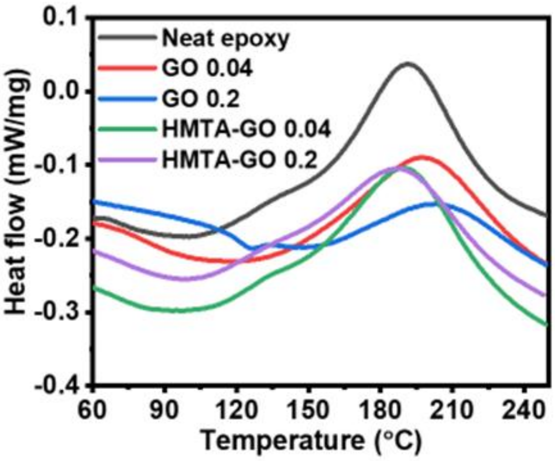

Figure S7. DSC curves for epoxy composites.

Reference

1. Xu, J.; Yang, J.; Liu, X.; Wang, H.; Zhang, J.; Fu, S. Preparation and characterization of fast-curing powder epoxy adhesive at middle temperature. *Royal Society open science* **2018**, *5*, 180566.

© 2019 by the authors. Licensee MDPI, Basel, Switzerland. This article is an open access article distributed under the terms and conditions of the Creative Commons Attribution (CC BY) license (http://creativecommons.org/licenses/by/4.0/).
